# Left ventricular remodeling after acute myocardial infarction: the influence of viability and revascularization - an echocardiographic substudy of the VIAMI-trial

**DOI:** 10.1186/1745-6215-15-329

**Published:** 2014-08-18

**Authors:** Ramon B van Loon, Gerrit Veen, Otto Kamp, Leo HB Baur, Albert C van Rossum

**Affiliations:** Department of Cardiology, 5F003, VU University Medical Center, De Boelelaan 1117, 1081 HV Amsterdam, The Netherlands; Department of Cardiology, Atrium Medical Center Parkstad, 6419 PC Heerlen and Faculty of Health, Medicine and Life Sciences, University Maastricht, 6200 MD Maastricht, The Netherlands

**Keywords:** Myocardial infarction, Viability, Echocardiography, Percutaneous coronary intervention, Remodeling

## Abstract

**Background:**

Viability seems to be important in preventing ventricular remodeling after acute myocardial infarction (AMI). We investigated the influence of viability, as demonstrated with low-dose dobutamine echocardiography, and the role of early revascularization on the process of left ventricular (LV) remodeling after AMI.

**Methods:**

We retrospectively investigated 224 patients who were initially included in the viability-guided angioplasty after acute myocardial infarction-trial (VIAMI-trial)*.* Patients in the VIAMI-trial did not undergo a primary or rescue percutaneous coronary intervention and were stable in the early in-hospital phase. Patients underwent viability testing within 72 hours after AMI. Patients with viability were randomized to an invasive strategy or an ischemia-guided strategy. Follow-up echocardiography was performed at a mean of 205 days. In this echocardiographic substudy, patients were divided into three new groups: group 1, viable and revascularized before follow-up echocardiogram; group 2, viable, but medically treated; and group 3, non-viable patients.

**Results:**

Group 1 showed preservation of LV volume indices. The ejection fraction (EF) increased significantly from 54.0% to 57.5% (*P* = 0.047). Group 2 showed a significant increase in LV volume indices with no improvement in EF (53.3% versus 53.0%, *P* = 0.86). Group 3 showed a significant increase in LV volume indices, with a decrease in EF from 53.5% to 49.1% (*P* = 0.043). Multivariate logistic regression analysis indicated the number of viable segments and revascularization during follow-up as independent predictors for EF improvement, especially in patients with lower EF at baseline.

**Conclusion:**

Viability early after AMI is associated with improvement in LV function after revascularization. When viable myocardium is not revascularized, the LV tends to remodel with increased LV volumes, without improvement of EF. Absence of viability results in ventricular dilatation and deterioration of EF, irrespective of revascularization status.

**Trial registration:**

NCT00149591 (assigned: 6 September 2005).

**Electronic supplementary material:**

The online version of this article (doi:10.1186/1745-6215-15-329) contains supplementary material, which is available to authorized users.

## Background

Following acute myocardial infarction (AMI) the development of left ventricular (LV) dilatation, caused by alterations in architecture and function of the left ventricle, is one of the most feared consequences of the complex process of ventricular remodeling. Ventricular remodeling involves both the infarcted and noninfarcted zone, and is considered as one of the major determinants of poor outcome [1]. Gaudron and colleagues showed that predictors of progressive LV dilatation and chronic LV dysfunction include ventriculographic LV size, LV ejection fraction (EF) at day 4 after AMI, infarct location (especially anterior), and Thrombolysis In Myocardial Infarction (TIMI) flow grade of the infarct-related artery (IRA) [2]; also, persistent occlusion of the IRA has been indicated as a predictor for ventricular remodeling [3–5]. The relative contribution of all these factors in the remodeling process remains unclear, but the patency of the IRA assumes particular importance because it is a risk factor amenable for intervention. Reperfusion therapy has contributed to an important reduction in mortality after AMI by limiting myocardial necrosis and therefore preservation of LV function.

### Viability and remodeling

The transmural extent of myocardial necrosis and the presence or absence of myocardial viability are important predictors for the onset of ventricular remodeling. Furthermore, the extent of microvascular obstruction as detected by cardiovascular magnetic resonance (CMR) influences the functional recovery after AMI [6].

At rest, most of the LV wall thickening reflects thickening of the inner layer of the myocardium. The middle layer contributes only moderately to overall wall thickening, while the outer third contributes only minimally [7]. Although the middle and outer layers of the myocardium thicken little at rest, they thicken more with catecholamine stimulation and thus contribute to overall wall thickening during dobutamine infusion [8]. Dobutamine responsiveness with wall thickening may therefore indicate the presence of viable myocardium in the middle and outer layers of the ventricular wall. An experimental study by Hochman and Bulkley indicated that only a small rim of viable epicardial myocardium prevented infarct expansion [9].

Older necropsy studies have already shown that the degree of transmurality was an important predictor of infarct expansion [10]. Later on, this concept of remodeling and transmurality was confirmed by CMR studies [11, 12]. Viability in the infarct zone, generally located in the outer layers of the ventricular wall, prevents remodeling by limiting infarct expansion. However, myocardial viability may only be temporarily present if no revascularization takes place. Knudsen and colleagues demonstrated a time-dependent loss of viability during the first 3 months after AMI [11].

Many studies showed a favorable effect of post-infarction viability on LV function and volume parameters. These effects were demonstrated in a population after AMI with successful percutaneous coronary intervention (PCI) or treatment with thrombolysis [13–15].

Viability can be detected with several imaging modalities. Low-dose dobutamine echocardiography (LDDE) is considered to be the test of first choice in patients with recent AMI, as it is a bedside tool with a diagnostic accuracy of about 80%, which is comparable to scintigraphical techniques (single photon emission computed tomography/positron emission tomography), and can be performed easily and safely 24 hours after AMI [16, 17]. Nowadays, CMR with late gadolinium enhancement reveals better diagnostic accuracy, even in patients with severe LV dysfunction, but lacks the usefulness of a bedside tool [12, 18].

The aim of this study is to investigate the influence of viability, demonstrated with LDDE, and the role of early revascularization on the process of LV remodeling after AMI.

## Methods

### Patients and protocol

We retrospectively studied patients who were initially included in the viability-guided angioplasty after acute myocardial infarction-trial (VIAMI-trial; NCT00149591: http://www.clinicaltrials.gov). The VIAMI-trial was a prospective, multicenter, randomized controlled clinical trial [19, 20]. Between April 2001 and January 2006, 291 patients were enrolled from 11 participating Dutch hospitals. Patients admitted to any of the participating centers with a (sub)acute myocardial infarction, who were not treated by primary or rescue angioplasty, and who were stable during the first 48 hours after the acute event, were screened for the study. Stable patients revealed no signs of ongoing ischemia based on electrocardiographic characteristics or persistent chest discomfort.

Patients less than 80 years of age were considered suitable for the study when they met the criteria for definite myocardial infarction: a significant rise in creatine kinase-MB levels (twice the upper limit of normal), 1 mm ST segment elevation in two or more standard leads or 2 mm ST segment elevation in two contiguous chest leads, and/or the development of Q waves.

Patients underwent LDDE for the detection of viability within 72 hours after AMI. Patients with viability in the infarct area were randomized to an invasive or a conservative treatment strategy. The invasive strategy patients underwent in-hospital coronary angiography with the intention to perform PCI with stenting of the IRA. In the conservative group, an ischemia-guided approach was adopted with stress testing before hospital discharge. After a positive test for ischemia, coronary angiography was strongly recommended.

Patients were followed up for 3 years, with the intension to perform an echocardiogram after 3, 6, and 12 months. Eventually, 224 patients had an evaluable baseline and follow-up echocardiogram.

In this echocardiographic substudy, patients were divided into three new groups to investigate the influence of revascularization and viability on LV remodeling. These groups were based on their initial randomization, LDDE response, and revascularization status. Ninety four patients were classified as viable and were revascularized before their follow-up echocardiogram (group 1). Seventy one patients were also viable, but medically treated without revascularization (group 2). Fifty nine patients were classified as non-viable (group 3).

### Ethical approval and consent

The VIAMI-trial was approved by the Clinical Research Ethical Committee of the VU University Medical Center (ref: 1999/123). The local research ethics committee of each participating hospital approved for local feasibility (see Additional file 1). All eligible patients provided written informed consent. The study complied with the Declaration of Helsinki.

### Echocardiographic examination

The baseline, LDDE, and follow-up echocardiograms were performed with a Hewlett-Packard Sonos 5500 imaging system (2.5 and 3.5 MHz transducers; Hewlett-Packard Inc., Andover, MA, USA). All patients were imaged while taking their prescribed medication. Beta-blockers were withdrawn 24 hours before the LDDE. At baseline and at follow-up, a complete cross sectional ultrasound examination was performed according to the guidelines of the American Society of Echocardiography under resting conditions [21].

To perform the LDDE, dobutamine was administrated intravenously at doses of 5, 10, and 15 μg/kg/min, for 5 minutes at each dose. When a 10% increase in heart rate was not achieved with 15 μg/kg/min, a 5-minute infusion with 20 μg/kg/min could be used as the final stage of the procedure [22–24]. Patients were continuously monitored by a 12-lead electrocardiogram, and blood pressure was recorded at the end of each stage. The criteria for stopping dobutamine infusion were as follows: hypotension decrease in systolic or diastolic blood pressure of more than 30 mmHg; hypertension (systolic blood pressure above 220 mmHg, diastolic pressure above 130 mmHg); intolerable angina; supraventricular tachycardia; ventricular tachycardia, significant ST segment depression or elevation (more than 2 mm); significant ischemia on the echocardiogram (more than 1 segment). All patients reached the 10% increase in heart rate. Patients with significant ischemia were excluded from the study because coronary angiography was mandatory.

All echocardiographic images were sent to the core-lab (VU University Medical Center) and analyzed by two experienced observers. A third observer was used in case of disagreement to reach consensus.

### Echocardiographic measurements

#### Baseline and follow-up

Five standard views were obtained: the parasternal long-axis and short-axis view (mid-ventricular view) and the apical two-, three- and four-chamber view. A 16-segment model was used in which the apex is divided into four segments. Segmental wall motion and thickening was scored according to a 4-point scale: 1 = normal, 2 = hypokinetic, 3 = akinetic, and 4 = dyskinetic. Wall motion score index was calculated by summing the scores for each segment and dividing by the number of segments analyzed. LV volumes and EF were measured by use of the modified Simpson’s rule algorithm from orthogonal apical long-axis projections (four- and two-chamber view) as recommended by the American Society of Echocardiography [21]. Tracing of the endocardial borders was performed on a digitized frame. The mean values of at least three measurements of the technically best cardiac cycles were taken from each examination. EF was calculated as:

$$\left[\mathrm{End}\;\mathrm{diastolic}\;\mathrm{volume}–\mathrm{end}\;\mathrm{systolic}\;\mathrm{volume}\right]/\mathrm{end}\;\mathrm{diastolic}\;\mathrm{volume}\;\times \;100.$$

A relative increase of LV ejection fraction >10% at follow-up was defined as a significant LV improvement [14].

#### Low-dose dobutamine echocardiogram

Viability was defined as the improvement of wall motion abnormalities in two or more segments of the infarct zone. Changes from hypokinesia to normokinesia and from dyskinesia or akinesia to hypo- or normokinesia are considered an improvement in wall motion abnormality. Dyskinesia changing to akinesia was not considered as an improvement.

### Statistical analysis

Baseline descriptive data are presented as mean ± SD. Differences in clinical and echocardiographic variables were assessed by unpaired Student’s *t* test. Differences between proportions were assessed by chi-square analysis; a Fisher’s exact test was used when appropriate. The LV end-diastolic volume (LVEDV) and end-systolic volume (LVESV) and EF at baseline and at follow-up were compared for mean values and changes over time, using one-way repeated measures analysis of variance, with time being the within-subject variable. Variables that were significantly different between patients with and without LVEF improvement (relative increase of >10%) were submitted for univariate regression analysis. Variables that showed a significant correlation with LVEF improvement were included in the multivariate stepwise logistic regression model to determine the independent correlates. A probability value of *P* < 0.05 was considered significant. All analyses were performed with the use of SPSS software, version 16.0 (SPSS, Inc., Chicago, IL, USA).

## Results

Of the 224 patients with an evaluable baseline and follow-up echocardiogram, 83 were randomized to the viable invasive strategy and 82 to the viable conservative approach. The remaining 59 patients had no viability and were followed in the registry group. In the viable invasive group, 13 patients did not undergo a revascularization procedure for different reasons (small vessels, multi-vessel disease without coronary artery bypass graft options, non-significant stenosis IRA). In the viable conservative group, 24 patients crossed over to the invasive group because of anginal complaints or proven ischemia before follow-up echocardiography. For the purpose of this echocardiographic substudy investigating the influence of revascularization and viability on LV remodeling, we divided the patients into three new groups: patients with viability and revascularization before their follow-up echocardiogram (94 patients, group 1); patients with viability but medically treated without revascularization before their follow-up echocardiogram (71 patients, group 2); and patients without viability (59 patients, group 3).

### Patient characteristics

Baseline characteristics of the three groups are shown in Table 1. Most baseline characteristics are similar, except for history of angina and PCI, current smoking, and use of statins. Furthermore, group 3 reveals more pathological Q-waves and more persistent ST segment elevation >1 mm on the electrocardiogram 48 hours after admission. Enzymatic and echocardiographic indicators of infarct size were comparable between the three groups at baseline.

**Table 1:** **Baseline characteristics and echocardiographic data**

	Viable		Non-viable	
	Revascularized	Medical		
Characteristic	Group 1 (n = 94)	Group 2 (n = 71)	Group 3 (n = 59)	***P*** value*
Male	83%	77%	69%	0.61
Age (years)	61	60	63	0.18
**Clinical history**				
Angina	43%	41%	56%	<0.001
Myocardial infarction	7%	3%	12%	0.056
Percutaneous coronary intervention	1%	3%	12%	0.013
Coronary artery bypass graft	0%	0%	0%	-
**In-hospital**				
Anterior infarction	29%	31%	46%	0.28
Thrombolysis	56%	49%	47%	0.83
Revascularization	100%	0%	29%	<0.001
- Percutaneous coronary intervention	83%	0%	24%	
- Coronary artery bypass graft	17%	0%	5%	
Peak creatine kinase (U/L)	1,617 ± 1,215	1,804 ± 1,321	1,784 ± 1,654	0.66
Peak creatine kinase-MB (U/L)	165 ± 130	204 ± 182	170 ± 149	0.27
Pathological Q waves (n)^†^	1.70 ± 1.57	1.96 ± 1.48	2.54 ± 2.00	0.01
Persistent ST elevation >1 mm^†^	20%	34%	54%	<0.001
**Echocardiography**				
Viable segments (16 segments model) (n)	3.39 ± 1.43	3.38 ± 1.27	0.37 ± 0.49	0.95^‡^
LVEDV (ml)	98.5 ± 33.2	88.4 ± 25.2	89.5 ± 33.7	0.07
LVESV (ml)	47.0 ± 25.1	41.9 ± 19.1	42.9 ± 22.5	0.32
LVEF	54.0%	53.3%	53.5%	0.93
WMI	1.54 ± 0.27	1.57 ± 0.26	1.54 ± 0.30	0.78
**Follow-up**				
Follow-up echocardiogram (days)	226 ± 129	178 ± 127	205 ± 134	0.07
Revascularisation (days)	25 ± 66	NA	48 ± 75	0.16^§^
**Risk factors**				
Diabetes mellitus	10%	11%	8%	0.63
Hypertension	23%	26%	36%	0.11
Hypercholesterolemia	19%	14%	17%	0.91
Current cigarette smoking	46%	35%	64%	0.003
Family history of coronary artery disease	33%	26%	20%	0.25
**Medications at admission**				
Aspirin	14%	11%	12%	0.76
Beta-blocker	11%	12%	17%	0.58
Statins	17%	5%	10%	0.02
ACE-inhibitor	10%	5%	15%	0.11
Angiotensin II antagonist	4%	6%	7%	0.33
**Medications at discharge**				
Aspirin	95%	99%	88%	0.04
Beta-blocker	97%	93%	92%	0.35
Statins	94%	99%	92%	0.18
ACE-inhibitor	54%	58%	68%	0.25
Angiotensin II antagonist	3%	7%	8%	0.35
Clopidogrel	65%	11%	22%	<0.001
Diuretics	5%	6%	17%	0.02

*Differences between the groups. ^†^On the electrocardiogram at 48 hours after acute myocardial infarction. ^§^Difference between group 1 and 2. Values are shown as means ± SD unless otherwise indicated. ACE, angiotensin converting enzyme; LVEDV, left ventricular end-diastolic volume; LVEF, left ventricular ejection fraction; LVESV, left ventricular end-systolic volume; NA, not applicable; WMI, wall motion score index.

### Left ventricular volumes and ejection fraction

**Figure 1:**
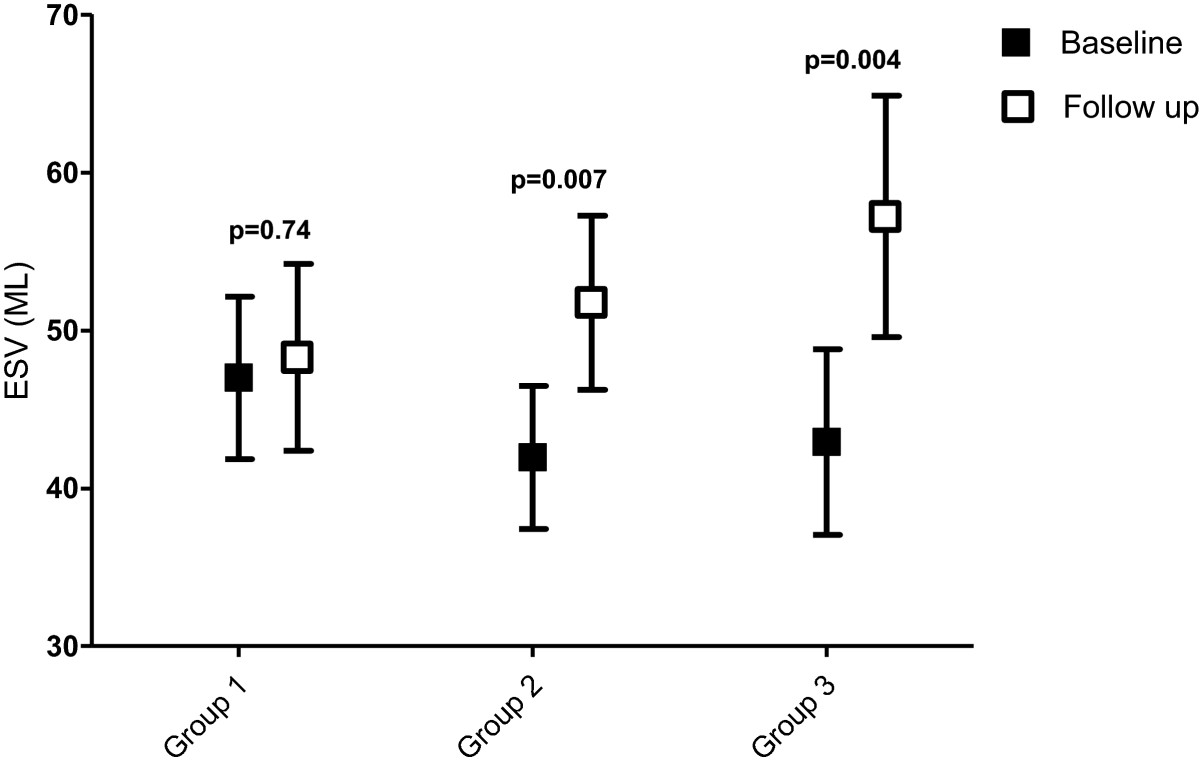
**Changes in end-systolic volumes.** Change in left ventricular end-systolic volume (ESV) between baseline and follow-up in groups 1 to 3.

**Figure 2:**
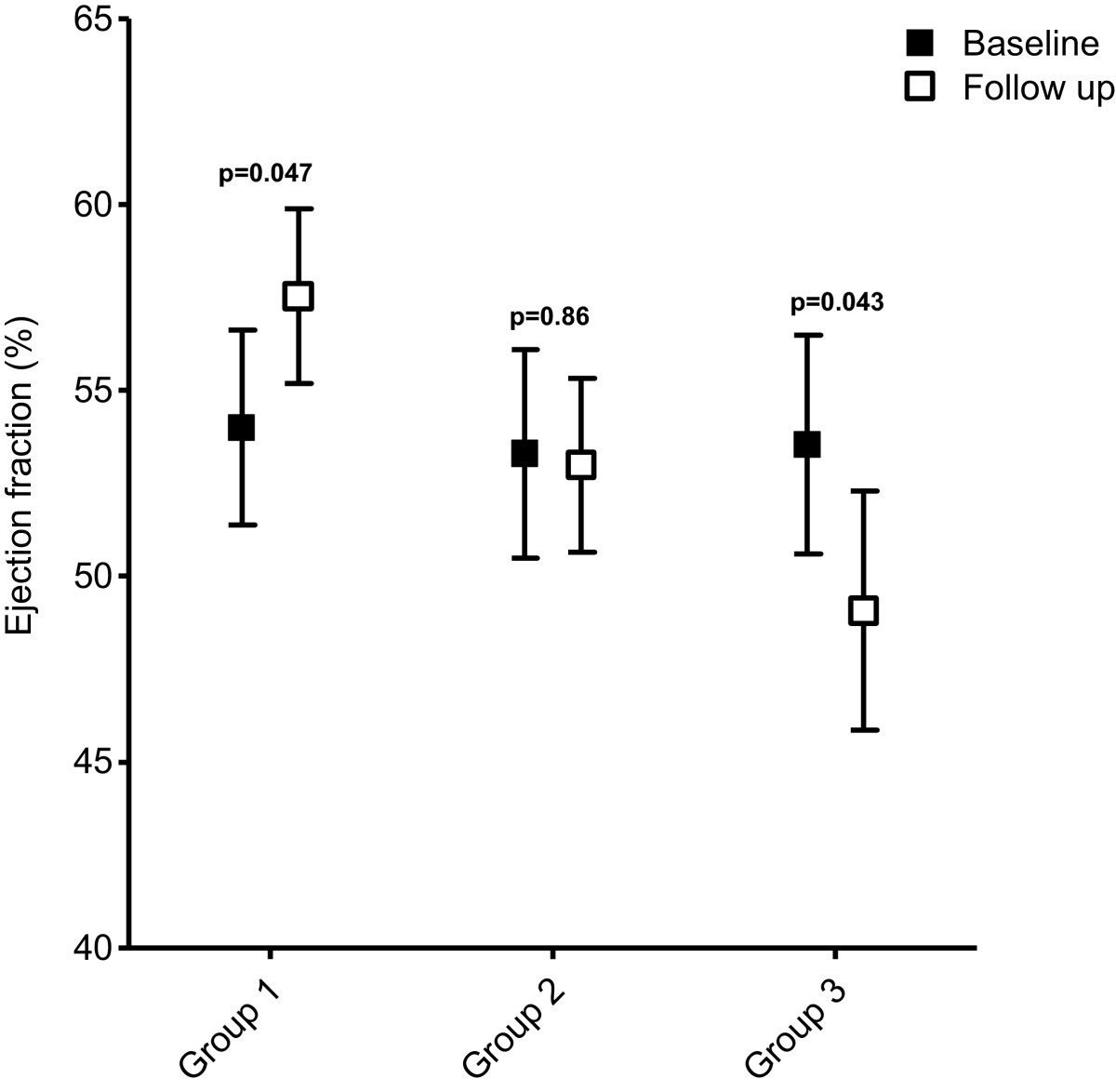
**Changes in ejection fraction.** Change in ejection fraction between baseline and follow-up in groups 1 to 3.

The outcome in the three patient groups is shown in Table 2 and Figures 1 and 2.

**Table 2:** **Baseline and follow-up left ventricular volumes and ejection fraction**

	LVEDV (ml)			LVESV (ml)			EF (%)		
	Baseline	Follow-up	***P*** value	Baseline	Follow-up	***P*** value	Baseline	Follow-up	***P*** value
Group 1	98.5 (33.2)	107.7 (39.3)	0.083	47.0 (25.1)	48.3 (28.9)	0.74	54.0 (12.8)	57.5 (11.5)	0.047
Group 2	88.4 (25.2)	106.5 (29.9)	0.001	41.9 (19.1)	51.7 (23.2)	0.007	53.3 (11.8)	53.0 (9.9)	0.86
Group 3	89.5 (33.7)	108.6 (32.9)	0.002	42.9 (22.5)	57.2 (29.3)	0.004	53.5 (11.3)	49.1 (12.3)	0.043

Values are mean (standard deviation). *P* values represent differences between baseline and follow-up. EF, ejection fraction; LVEDV, left ventricular end-diastolic volume; LVESV, left ventricular end-systolic volume.

In patients without viability (group 3) a significant increase in LVEDV and LVESV was observed at follow-up, with a decrease in LVEF from 53.5% to 49.1% (*P* = 0.043). Patients with viability who were medically treated during their follow-up period (group 2) showed a significant increase in LVEDV and LVESV without a change in LVEF (53.3% versus 53.0%, *P* = 0.86). In contrast, patients with viability who were revascularized during follow-up (group 1) showed a non-significant increase in LVEDV, with no change in LVESV. The LVEF increased significantly from 54.0% to 57.5% (*P* = 0.047).

**Figure 3:**
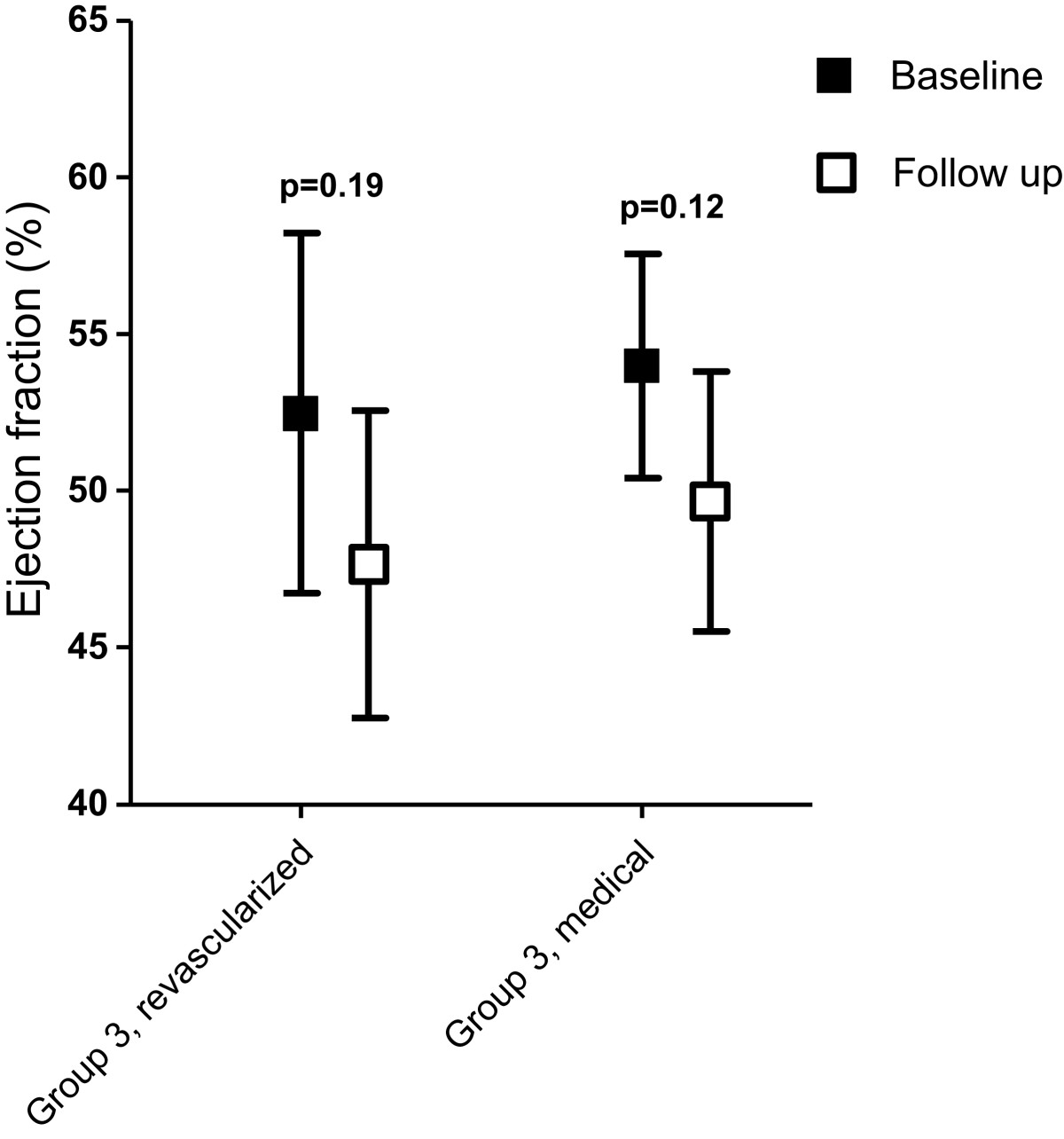
**Changes in ejection fraction in group 3 after revascularization or medical treatment.** Change in ejection fraction between baseline and follow-up in non-viable invasive-treated versus non-viable medically treated group (group 3).

In patients without viability (group 3), revascularization did not lead to improvement in LVEF or volumes. Both the revascularized (n = 17) and the medically treated (n = 42) patients in the non-viable group showed a significant increase in LVEDV and LVESV. A non-significant decrease in EF is observed in both subgroups (Figure 3 and Table 3).

**Table 3:** **Baseline and follow-up left ventricular volumes and ejection fraction in group 3**

	LVEDV (ml)			LVESV (ml)			EF (%)		
	Baseline	Follow-up	***P*** value	Baseline	Follow-up	***P*** value	Baseline	Follow-up	***P*** value
Group 3 (total)	89.5 (33.7)	108.6 (32.9)	0.002	42.9 (22.5)	57.2 (29.3)	0.004	53.5 (11.3)	49.1 (12.3)	0.043
Group 3 (revascularized)	92.1 (35.1)	117.4 (29.4)	0.030	44.9 (23.7)	62.5 (21.6)	0.030	52.5 (11.2)	47.6 (9.5)	0.185
Group 3 (medically treated)	88.4 (33.6)	105.0 (33.9)	0.027	42.1 (22.3)	55.1 (31.9)	0.034	54.0 (11.4)	49.7 (13.3)	0.115

Values are mean (standard deviation). *P* values represent differences between baseline and follow-up. EF, ejection fraction; LVEDV, left ventricular end-diastolic volume; LVESV, left ventricular end-systolic volume.

### Predictors of left ventricular ejection fraction improvement

**Figure 4:**
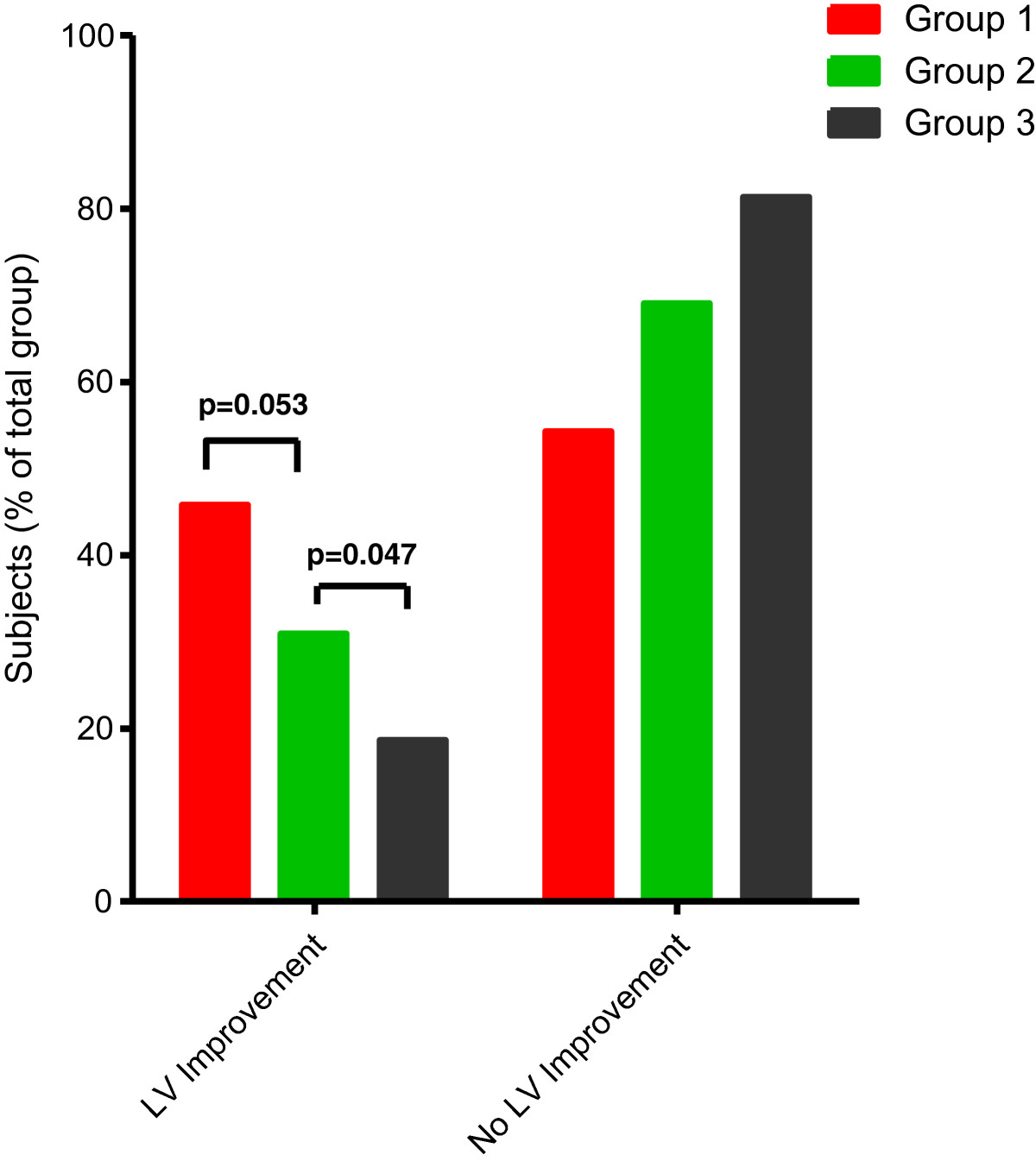
**Left ventricular improvement.** Percentage of significant left ventricular (LV) improvement in groups 1 to 3.

A significant improvement in LVEF (>10%) occurred in 46% of group 1 versus 31% of group 2 and 19% of group 3 patients (Figure 4). In Table 4 patients were divided into those with and those without LVEF improvement. The baseline characteristics are comparable. The group with improvement of LVEF underwent significantly more revascularization procedures (62 versus 43%, *P* = 0.008). Furthermore, more viable segments during baseline LDDE were seen (3.2 versus 2.3, *P* < 0.001) in this patient group. Also, the LV volume indices were significantly higher at baseline with a lower EF (45.9% versus 57.7%, *P* < 0.001). In the univariate analysis, all these parameters were found to be predictive for LVEF improvement. After multivariate logistic regression analysis was performed, three variables emerged as independent predictors of LVEF improvement: revascularization procedure before follow-up echocardiogram, number of viable segments and wall motion score index at baseline (Table 5).

**Table 4:** **Baseline characteristics and echocardiographic data of patients with or without a significant improvement in LVEF (>10%) at follow-up**

Characteristic	No improvement (n = 148)	Improvement (n = 76)	***P*** value*
Male	80%	70%	0.09
Age (years)	61	61	0.75
**Clinical history**			
Angina	44%	49%	0.50
Myocardial infarction	7%	7%	0.96
Percutaneous coronary intervention	5%	5%	0.86
Coronary artery bypass graft	0%	0%	-
**In-hospital and follow-up**			
Anterior infarction	38%	39%	0.81
Thrombolysis	51%	51%	0.93
Peak creatine kinase (U/L)	1802 ± 1439	1567 ± 1245	0.22
Peak creatine kinase-MB (U/L)	185 ± 150	166 ± 159	0.39
Pathological Q waves (n)^†^	2.1 ± 1.76	1.9 ± 1.58	0.53
Persistent ST elevation > 1 mm^†^	34%	32%	0.67
Revascularization before follow-up	43%	62%	0.008
**Echocardiography**			
Viable segments (16 segments model) (n)	2.3 ± 1.71	3.2 ± 1.78	<0.001
LVEDV (ml)	89.34 ± 32.44	99.84 ± 27.78	0.012
LVESV (ml)	38.91 ± 21.12	54.84 ± 22.03	<0.001
LV EF (%)	57.66 ± 10.93	45.87 ± 10.31	<0.001
WMI	1.51 ± 0.25	1.62 ± 0.31	0.01
follow-up echocardiogram (days)	208 ± 133	201 ± 127	0.70
**Risk factors**			
Diabetes mellitus	9%	12%	0.50
Hypertension	29%	24%	0.35
Hypercholesterolemia	16%	17%	0.83
Current cigarette smoking	46%	47%	0.91
Family history of coronary artery disease	26%	29%	0.61
**Medications at admission**			
Aspirin	12%	13%	0.74
Beta-blocker	13%	13%	0.96
Statins	10%	12%	0.60
ACE-inhibitor	11%	7%	0.27
Angiotensin II antagonist	7%	3%	0.09
**Medications at discharge**			
Aspirin	94%	95%	0.80
Beta-blocker	95%	93%	0.73
Statins	95%	95%	0.96
ACE-inhibitor	57%	63%	0.36
Angiotensin II antagonist	6%	5%	0.80
Clopidogrel	32%	45%	0.08
Diuretics	8%	9%	0.79

*Differences between the groups. ^†^On the electrocardiogram at 48 hours after acute myocardial infarction. Values are shown as means ± SD unless otherwise indicated. ACE, angiotensin converting enzyme; LVEDV, left ventricular end-diastolic volume; LVEF, left ventricular ejection fraction; LVESV, left ventricular end-systolic volume; WMI, wall motion score index.

**Table 5:** **Logistic regression analysis: predictors at baseline of left ventricular ejection fraction improvement**

	Univariate		Multivariate	
Variable	Wald ***χ*** ^2^	***P*** value	Wald ***χ*** ^2^	***P*** value
Revascularization before follow-up	6.84	0.009	5.74	0.017
Number of viable segments	12.84	>0.001	10.31	0.001
LVEDV	5.43	0.02	-	-
LVESV	19.57	>0.001	-	-
WMI	7.22	0.007	6.22	0.013

LVEDV, left ventricular end-diastolic volume; LVEF, left ventricular ejection fraction; LVESV, left ventricular end-systolic volume; WMI, wall motion score index.

### Inter-observer and intra-observer reproducibility

There was low variability (percentage difference in values) between LV volume measurements made by two independent observers (inter-observer variability). A sample of 25 patients per group was randomly selected and re-analyzed. Inter-observer variability was 7.25% with a good intra-class correlation coefficient of 0.86.

The LDDEs were analyzed by two experienced observers. There was low inter-observer variability in the classification of wall motion and the response to low-dose dobutamine (agreement 96%). In only 4% of the LDDE was a third observer used to reach consensus.

## Discussion

Our study shows the importance of revascularization in patients with viability early after AMI. To the best of our knowledge this is the largest study investigating the influence of viability and revascularization on LV remodeling in a patient group treated without primary or rescue PCI.

### Viability and revascularization

Bolognese and colleagues were the first to demonstrate the importance of myocardial viability to prevent LV dilatation in a population with successful PCI for the treatment of AMI [15]. However, only the influence of viability in patients with an open artery was evaluated. Kim and Braunwald, in the “open artery hypothesis”, proposed that, in addition to time-dependent myocardial salvage, beneficial effects of reperfusion therapy include attenuation of LV remodeling and promotion of electrical stability as an independent effect of an open IRA [25]. Rizzello and colleagues demonstrated only functional recovery after revascularization in segments with viable myocardium (at low-dose dobutamine) in patients with ischemic cardiomyopathy [26]. Coletta and colleagues showed that viable myocardium within the infarct zone (contractile reserve at low-dose dobutamine at 8 days after anterior myocardial infarction) obviates LV remodeling [27]. Bellenger and colleagues provided evidence that coronary recanalization, even late after AMI (between 3 days and 6 weeks; mean 26 days), can lead to attenuation of subsequent LV remodeling. A subset of patients (n = 26) from the open artery trial (TOAT) was studied. Dobutamine-stress CMR was used to assess myocardial viability in patients with anterior myocardial infarction, LV dysfunction, and isolated proximal occlusion of the left anterior descending coronary artery (LAD), who either underwent late PCI with a stent to the LAD, or medical treatment alone. The study demonstrated a significant relation between the number of viable segments within the infarct zone and improvement in end-systolic volume index and EF after revascularization [28]. Furthermore, a nuclear substudy of the occluded artery trial (NUC-OAT) investigated the influence of infarct-zone viability on ventricular remodeling after PCI or optimal medical treatment alone in a subsequent group of 124 patients [29]. The data from this study showed that there was no influence of infarct-zone viability on ventricular remodeling after PCI compared to medical therapy alone in patients with a total occlusion of the IRA.

Although there are some conflicting data upon this topic, most data support the theory that full patency of the IRA and viability are important for reducing both infarct expansion in the early phase of infarction and LV enlargement later on.

In our study, revascularization and the number of viable segments demonstrated by LDDE were both strong predictors for LVEF improvement at follow-up. The presence of viability alone was not enough to improve LV function. Probably, without revascularization a significant amount of myocardial viability disappears lacking optimal recanalization during the follow-up period, as already demonstrated by Knudsen and colleagues [11]. Furthermore, maximal yield of revascularization in patients with viability to improve LVEF was seen in patients with lower EF at baseline. These patients had more viable segments with the ability to regain function over time with improvement of LV function. However, even in patients with preserved LV function after AMI and only two viable segments (stunned myocardium), revascularization remained an independent predictor for further LVEF improvement.

### Study limitations

Because this is a large retrospectively studied population, multiple biases could have been of influence. In the VIAMI-trial, patients were randomized to medical treatment or an invasive strategy of coronary revascularization. Some of the patients in both groups were not treated as the protocol prescribed. For the purpose of this echocardiographic substudy, we divided the patients into three new groups based on viability and revascularization status. Therefore, selection bias cannot be excluded; however, no differences in clinical baseline characteristics were found, making the three groups well comparable.

No standard angiography was performed in the medically treated patient group. As a consequence, no information about the severity of stenosis or patency of the IRA is available in this patient group. Therefore, the influence of viability on LV remodeling in relation to IRA patency or severity of stenosis was not investigated.

Another point of attention is the use of LDDE for the detection of viability. Although LDDE is a well validated and feasible bedside technique, it is prone to inter- and intra-observer variability. In our study all images were sent to a core-lab and scored by experienced cardiologists with low inter-observer variability. Furthermore, sufficient echo-windows for reliable measurements are seen in about 80% of a normal study population. This can be improved to 90 to 95% with the use of ultrasound contrast agents [30]. The quality of the images is operator-dependent. In our study images were recruited from 11 Dutch centers with many echocardiography technicians. This is one of the reasons for having only 224 patients (out of 291) with sufficiently evaluable echocardiographic images at baseline and follow-up.

Since this study was conducted between 2001 and 2006, part of the medical treatment does not apply to today’s standard. During the inclusion period of the VIAMI-trial, treatment with clopidogrel was not standard care in patients without stents. Standard treatment with clopidogrel according to current guidelines (CLARITY-TIMI 28 trial) could have made the differences less pronounced [31]. Also the use of angiotensin converting enzyme (ACE) inhibitors and angiotensin II antagonists is low at discharge. During the early days of our study ACE inhibitors were only given to patients with anterior infarction or LV systolic dysfunction. Our study population has predominantly a preserved LV function which is in part the explanation for the low use of ACE inhibitors. However, there is no significant difference in ACE inhibitor use between the three groups making the influence of ACE inhibition on the process of remodeling comparable.

## Conclusions

We studied patients with AMI who were not treated with primary or rescue PCI. Revascularization in the patients with viability in the early phase after AMI leads to a significant improvement in LV function whereas, without revascularization, LV volumes increase without change in EF. The absence of viability results in ventricular dilatation and deterioration of the LVEF (irrespective of revascularization status). The number of viable segments and revascularization during the follow-up period (mean 205 days) are independent predictors for LVEF improvement, especially in patients with lower EF at baseline.

These findings support the importance of a widely patent IRA in the presence of myocardial viability to improve LVEF and to prevent LV remodeling. Revascularization in patients without viability plays no role in the prevention of remodeling.

Furthermore, the results suggest that patients with a significant amount of viability should be revascularized irrespective of the presence or absence of anginal complaints or objective signs of ischemia.

## Electronic supplementary material

Additional file 1: Names of ethical bodies connected to the VIAMI-trial.(DOCX 12 KB)

Below are the links to the authors’ original submitted files for images.

Authors’ original file for figure 1

Authors’ original file for figure 2

Authors’ original file for figure 3

Authors’ original file for figure 4

## Abbreviations

ACE: angiotensin converting enzyme
AMI: acute myocardial infarction
CMR: cardiovascular magnetic resonance
EF: ejection fraction
IRA: infarct-related artery
LAD: left anterior descending coronary artery
LDDE: low-dose dobutamine echocardiography
LV: left ventricular
LVEDV: left ventricular end-diastolic volume
LVESV: left ventricular end-systolic volume
PCI: percutaneous coronary intervention
VIAMI-trial: viability-guided angioplasty after acute myocardial infarction-trial.

## References

[1] St John SM, Pfeffer MA, Plappert T, Rouleau JL, Moye LA, Dagenais GR, Lamas GA, Klein M, Sussex B, Goldman S (1994). Quantitative two-dimensional echocardiographic measurements are major predictors of adverse cardiovascular events after acute myocardial infarction. The protective effects of captopril. Circulation.

[2] Gaudron P, Eilles C, Kugler I, Ertl G (1993). Progressive left ventricular dysfunction and remodeling after myocardial infarction. Potential mechanisms and early predictors. Circulation.

[3] Golia G, Marino P, Rametta F, Nidasio GP, Prioli MA, Anselmi M, Destro G, Zardini P (1994). Reperfusion reduces left ventricular dilatation by preventing infarct expansion in the acute and chronic phases of myocardial infarction. Am Heart J.

[4] Jeremy RW, Hackworthy RA, Bautovich G, Hutton BF, Harris PJ (1987). Infarct artery perfusion and changes in left ventricular volume in the month after acute myocardial infarction. J Am Coll Cardiol.

[5] Warren SE, Royal HD, Markis JE, Grossman W, McKay RG (1988). Time course of left ventricular dilation after myocardial infarction: influence of infarct-related artery and success of coronary thrombolysis. J Am Coll Cardiol.

[6] Nijveldt R, Beek AM, Hirsch A, Stoel MG, Hofman MB, Umans VA, Algra PR, Twisk JW, van Rossum AC (2008). Functional recovery after acute myocardial infarction: comparison between angiography, electrocardiography, and cardiovascular magnetic resonance measures of microvascular injury. J Am Coll Cardiol.

[7] Myers JH, Stirling MC, Choy M, Buda AJ, Gallagher KP (1986). Direct measurement of inner and outer wall thickening dynamics with epicardial echocardiography. Circulation.

[8] Sklenar J, Ismail S, Villanueva FS, Goodman NC, Glasheen WP, Kaul S (1994). Dobutamine echocardiography for determining the extent of myocardial salvage after reperfusion. An experimental evaluation. Circulation.

[9] Hochman JS, Bulkley BH (1982). Expansion of acute myocardial infarction: an experimental study. Circulation.

[10] Pirolo JS, Hutchins GM, Moore GW (1986). Infarct expansion: pathologic analysis of 204 patients with a single myocardial infarct. J Am Coll Cardiol.

[11] Knudsen AS, Darwish AZ, Norgaard A, Gotzsche O, Thygesen K (1998). Time course of myocardial viability after acute myocardial infarction: an echocardiographic study. Am Heart J.

[12] Kim RJ, Wu E, Rafael A, Chen EL, Parker MA, Simonetti O, Klocke FJ, Bonow RO, Judd RM (2000). The use of contrast-enhanced magnetic resonance imaging to identify reversible myocardial dysfunction. N Engl J Med.

[13] Bolognese L, Neskovic AN, Parodi G, Cerisano G, Buonamici P, Santoro GM, Antoniucci D (2002). Left ventricular remodeling after primary coronary angioplasty: patterns of left ventricular dilation and long-term prognostic implications. Circulation.

[14] Nijland F, Kamp O, Verhorst PM, de Voogt WG, Bosch HG, Visser CA (2002). Myocardial viability: impact on left ventricular dilatation after acute myocardial infarction. Heart.

[15] Bolognese L, Cerisano G, Buonamici P, Santini A, Santoro GM, Antoniucci D, Fazzini PF (1997). Influence of infarct-zone viability on left ventricular remodeling after acute myocardial infarction. Circulation.

[16] Schinkel AF, Poldermans D, Elhendy A, Bax JJ (2007). Assessment of myocardial viability in patients with heart failure. J Nucl Med.

[17] Bax JJ, Wijns W, Cornel JH, Visser FC, Boersma E, Fioretti PM (1997). Accuracy of currently available techniques for prediction of functional recovery after revascularization in patients with left ventricular dysfunction due to chronic coronary artery disease: comparison of pooled data. J Am Coll Cardiol.

[18] Bondarenko O, Beek AM, Hofman MB, Kuhl HP, Twisk JW, van Dockum WG, Visser CA, van Rossum AC (2005). Standardizing the definition of hyperenhancement in the quantitative assessment of infarct size and myocardial viability using delayed contrast-enhanced CMR. J Cardiovasc Magn Reson.

[19] van Loon RB, Veen G, Kamp O, Bronzwaer JGF, Visser CA, Visser FC (2004). Early and long-term outcome of elective stenting of the infarct-related artery in patients with viability in the infarct-area: rationale and design of the viability-guided angioplasty after acute myocardial infarction-trial (the VIAMI-trial). Current Contr Trials Cardiovas Med.

[20] van Loon RB, Veen G, Baur LH, Kamp O, Bronzwaer JG, Twisk JW, Verheugt FW, van Rossum AC (2012). Improved clinical outcome after invasive management of patients with recent myocardial infarction and proven myocardial viability: primary results of a randomized controlled trial (VIAMI-trial). Trials.

[21] Schiller NB, Shah PM, Crawford M, DeMaria A, Devereux R, Feigenbaum H, Gutgesell H, Reichek N, Sahn D, Schnittger I (1989). Recommendations for quantitation of the left ventricle by two-dimensional echocardiography. American Society of Echocardiography Committee on Standards, subcommittee on quantitation of two-dimensional echocardiograms. J Am Soc Echocardiogr.

[22] Nijland F, Kamp O, Verhorst PM, de Voogt WG, Visser CA (2001). In-hospital and long-term prognostic value of viable myocardium detected by dobutamine echocardiography early after acute myocardial infarction and its relation to indicators of left ventricular systolic dysfunction. Am J Cardiol.

[23] Afridi I, Main ML, Grayburn PA (1996). Accuracy of dobutamine echocardiography for detection of myocardial viability in patients with an occluded left anterior descending coronary artery. J Am Coll Cardiol.

[24] Zaglavara T, Haaverstad R, Cumberledge B, Irvine T, Karvounis H, Parharidis G, Louridas G, Kenny A (2002). Dobutamine stress echocardiography for the detection of myocardial viability in patients with left ventricular dysfunction taking beta blockers: accuracy and optimal dose. Heart.

[25] Kim CB, Braunwald E (1993). Potential benefits of late reperfusion of infarcted myocardium. The open artery hypothesis. Circulation.

[26] Rizzello V, Poldermans D, Boersma E, Biagini E, Schinkel AF, Krenning B, Elhendy A, Vourvouri EC, Sozzi FB, Maat A, Crea F, Roelandt JR, Bax JJ (2004). Opposite patterns of left ventricular remodeling after coronary revascularization in patients with ischemic cardiomyopathy: role of myocardial viability. Circulation.

[27] Coletta C, Sestili A, Seccareccia F, Rambaldi R, Ricci R, Galati A, Bigi R, Aspromonte N, Renzi M, Ceci V (2003). Influence of contractile reserve and inducible ischaemia on left ventricular remodelling after acute myocardial infarction. Heart.

[28] Bellenger NG, Yousef Z, Rajappan K, Marber MS, Pennell DJ (2005). Infarct zone viability influences ventricular remodelling after late recanalisation of an occluded infarct related artery. Heart.

[29] Udelson JE, Pearte CA, Kimmelstiel CD, Kruk M, Kufera JA, Forman SA, Teresinska A, Bychowiec B, Marin-Neto JA, Hochtl T, Cohen EA, Caramori P, Busz-Papiez B, Adlbrecht C, Sadowski ZP, Ruzyllo W, Kinan DJ, Lamas GA, Hochman JS (2011). The occluded artery trial (OAT) viability ancillary study (OAT-NUC): influence of infarct zone viability on left ventricular remodeling after percutaneous coronary intervention versus optimal medical therapy alone. Am Heart J.

[30] Grayburn PA, Mulvagh S, Crouse L (2002). Left ventricular opacification at rest and during stress. Am J Cardiol.

[31] Sabatine MS, Cannon CP, Gibson CM, Lopez-Sendon JL, Montalescot G, Theroux P, Claeys MJ, Cools F, Hill KA, Skene AM, McCabe CH, Braunwald E (2005). Addition of clopidogrel to aspirin and fibrinolytic therapy for myocardial infarction with ST-segment elevation. N Engl J Med.

