# Supplementary material for: Left ventricular remodeling after acute myocardial infarction: the influence of viability and revascularization - an echocardiographic substudy of the VIAMI-trial
Source: Trials. 2014 Aug 18;15:329. doi: 10.1186/1745-6215-15-329 (PMC4141086; doi:10.1186/1745-6215-15-329)
Supplement: Supplementary file 1 — Additional file 1: Names of ethical bodies connected to the VIAMI-trial.(DOCX 12 KB) [file 13063_2014_2199_MOESM1_ESM.docx]

**Names ethical bodies VIAMI-trial**

- Medical ethical committee of the VU University Medical Center.
- Medical ethical committee of Atrium Medical Center Parkstad, Heerlen and Faculty of Health, Medicine and Life Sciences, University Maastricht.
- Medical ethical committee of Diakonessenhuis, Utrecht
- Medical ethical committee of the University Medical Center St. Radboud, Nijmegen.
- Medical ethical committee of Ziekenhuis Hilversum.
- Medical ethical committee of Kennemer Gasthuis, Haarlem.
- Medical ethical committee of Lucas-Andreas Ziekenhuis, Amsterdam.
- Medical ethical committee of Catharina Ziekenhuis, Eindhoven.
- Medical ethical committee Noord-Holland:

- Waterland Ziekenhuis, Purmerend.

- Zaans Medisch Centrum De Heel, Zaandam.

- Medisch Centrum Alkmaar, Alkmaar.
