# Supplementary figures and images for: Left ventricular remodeling after acute myocardial infarction: the influence of viability and revascularization - an echocardiographic substudy of the VIAMI-trial
Source: Trials. 2014 Aug 18;15:329. doi: 10.1186/1745-6215-15-329 (PMC4141086; doi:10.1186/1745-6215-15-329)

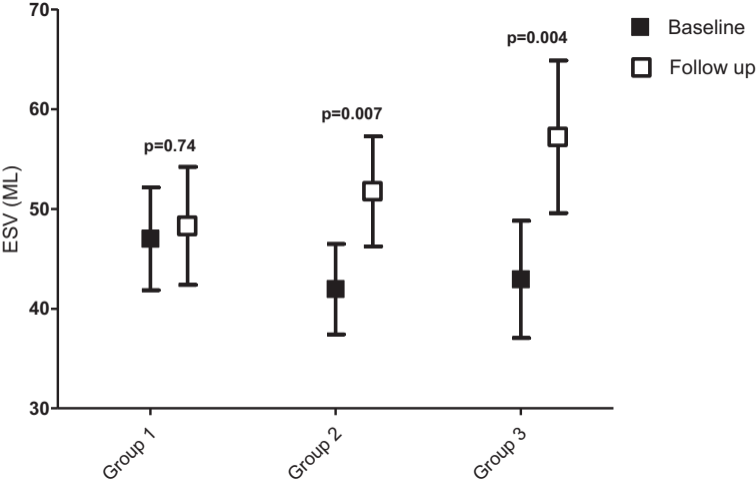

Supplement: Supplementary file 2 — Authors’ original file for figure 1 [file 13063_2014_2199_MOESM2_ESM.pdf]

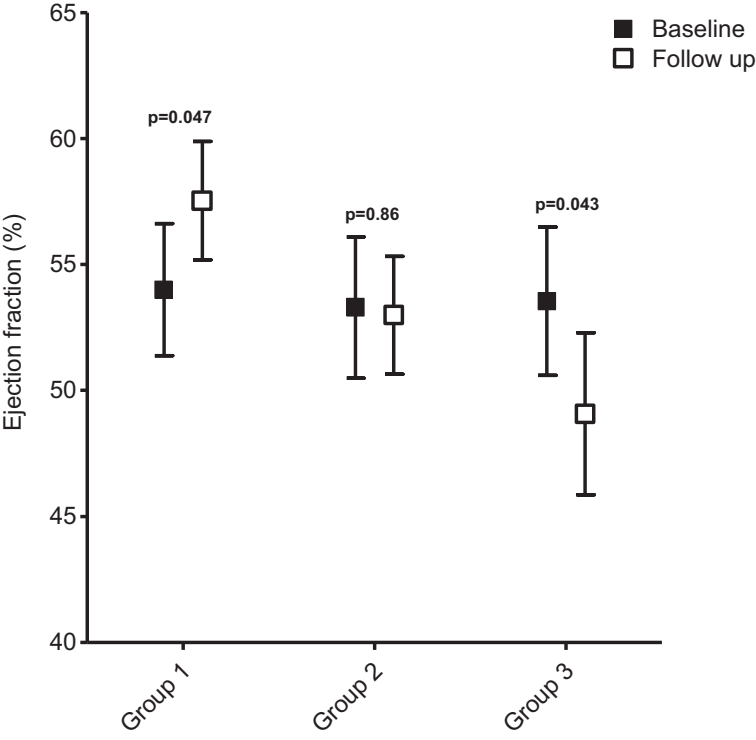

Supplement: Supplementary file 3 — Authors’ original file for figure 2 [file 13063_2014_2199_MOESM3_ESM.pdf]

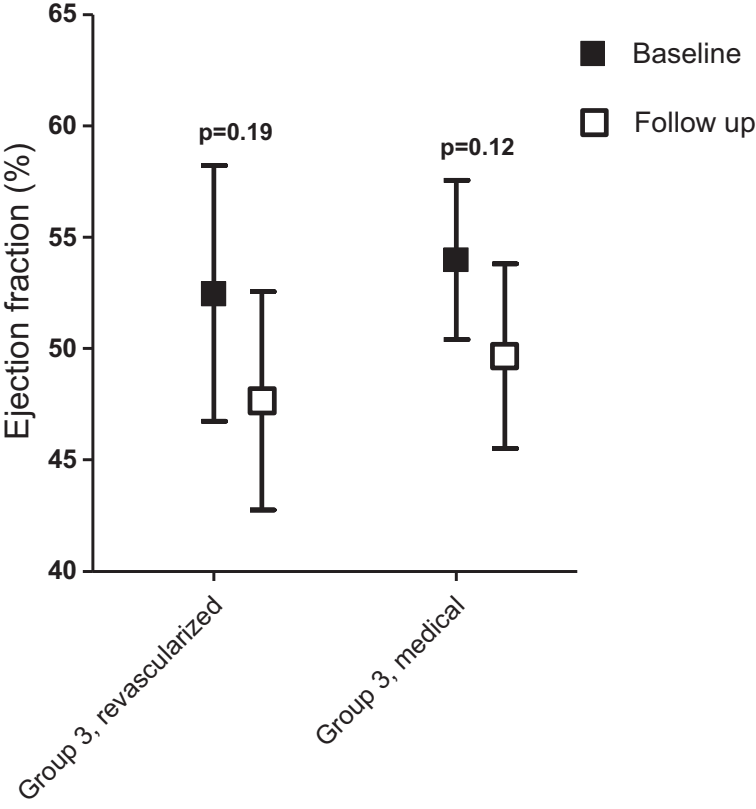

Supplement: Supplementary file 4 — Authors’ original file for figure 3 [file 13063_2014_2199_MOESM4_ESM.pdf]

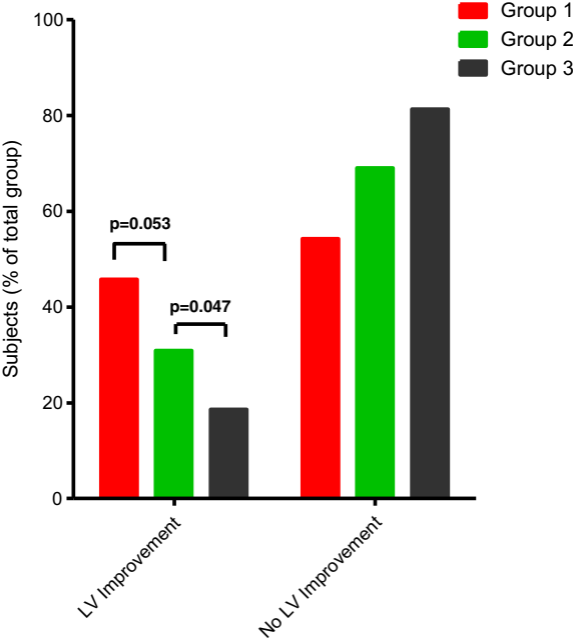

Supplement: Supplementary file 5 — Authors’ original file for figure 4 [file 13063_2014_2199_MOESM5_ESM.pdf]
